# Routine Echocardiographic Assessment in LVAD Patients—A Structured Approach to Acquisition and Interpretation

**DOI:** 10.3390/jcdd13020070

**Published:** 2026-01-30

**Authors:** Nicolas Merke, Felix Schoenrath, Evgenij Potapov, Jan Knierim

**Affiliations:** 1 Department of Cardiothoracic and Vascular Surgery, Deutsches Herzzentrum der Charité—Medical Heart Center of Charité and German Heart Institute Berlin, Augustenburger Platz 1, 13353 Berlin, Germany; nicolas.merke@dhzc-charite.de (N.M.); felix.schoenrath@dhzc-charite.de (F.S.); evgenij.potapov@dhzc-charite.de (E.P.); 2 Charité—Universitätsmedizin Berlin, Corporate Member of Freie Universität Berlin and Humboldt Universität zu Berlin, Charitéplatz 1, 10117 Berlin, Germany; 3 DZHK (German Centre for Cardiovascular Research), Partner Site Berlin, Hessische Straße 3-4, 10115 Berlin, Germany; 4 Department of Internal Medicine and Cardiology, Sana Paulinenkrankenhaus, Dickensweg 25-39, 14044 Berlin, Germany

**Keywords:** echocardiography, LVAD, assist, VAD, imaging, hemodynamics

## Abstract

Durable left ventricular assist devices (LVADs) are an established and highly effective therapy for patients with advanced heart failure. Ongoing technological improvements and structured follow-up programs have significantly enhanced device durability, reduced complications, and improved long-term survival. Consequently, a growing number of patients with LVAD support require long-term outpatient care and increasingly present to both specialized and non-specialized hospitals, including for admissions unrelated to heart failure. In this context, echocardiography plays a central role. It is essential not only for routine follow-up at dedicated LVAD clinics but also for the assessment of cardiac status during inpatient admissions for extracardiac conditions. However, echocardiographic evaluation in LVAD patients is technically demanding and requires a solid understanding of LVAD physiology, device–heart interactions, and the specific hemodynamic conditions of continuous-flow support. Without this knowledge, standard echocardiographic parameters may be misleading. This review provides sonographers and cardiologists with a practical, clinically oriented framework for routine transthoracic echocardiography in patients with durable LVAD support. We summarize key principles of LVAD hemodynamics, discuss interpretation of LVAD console parameters, propose a standardized imaging protocol, and outline a structured approach to common echocardiographic findings in routine ambulatory and inpatient settings.

## 1. Introduction

Mechanical circulatory support, whether as durable long-term therapy, bridge to transplantation, or bridge to recovery, has become an integral component in the management of advanced heart failure [1]. Over the past decade, substantial technological advances have markedly improved the durability and reliability of contemporary left ventricular assist devices (LVADs) [2]. Together with refined patient selection and the implementation of structured follow-up programs, these developments have led to a reduction in device-related complications and, consequently, to a significant improvement in long-term survival among patients supported with durable LVADs.

As a result, a growing number of patients supported by LVADs require long-term outpatient care and increasingly present not only to implanting centers but also to non-specialized hospitals and outpatient clinics with acute cardiac or extracardiac conditions. In these settings, urgent questions frequently arise regarding device function, cardiac performance, and hemodynamic stability. Echocardiography remains the most readily available and widely used imaging modality for assessing cardiac function and the interaction between the heart and the LVAD in this patient population.

Importantly, the vast majority of echocardiographic examinations in LVAD patients are performed in the context of routine ambulatory follow-up or during non-device-related hospital admissions. These examinations differ fundamentally from perioperative or early postoperative assessments and are typically conducted outside highly specialized LVAD programs. Accordingly, the focus is on standardized evaluation of cardiac structure, ventricular loading, valve function, and device–heart interaction under stable support conditions, rather than on complex decision-making scenarios such as assessment of myocardial recovery, candidacy for LVAD explantation, or planning of interventional treatment strategies (e.g., for aortic valve insufficiency).

However, echocardiography in LVAD patients is technically demanding. Standard imaging windows—particularly apical views—are frequently compromised by the presence of the inflow cannula at the LV apex and by device-related artifacts. As a result, conventional echocardiographic parameters may be difficult to acquire, poorly reproducible, or misleading if interpreted outside the specific hemodynamic context of mechanical circulatory support. Alternative imaging approaches, including off-axis parasternal views or transhepatic windows, have been proposed and may improve visualization of ventricular size and function, albeit often at the expense of temporal resolution.

Adjustment of LVAD support profoundly influences cardiac structure and function. Pump speed and loading conditions affect left ventricular unloading and reverse remodeling, aortic valve opening patterns, the development of clinically relevant aortic regurgitation, and right ventricular performance. In this setting, echocardiography is the primary diagnostic tool for longitudinal follow-up and plays a central role in guiding routine device optimization and medical therapy.

Given these challenges and the growing number of patients requiring longitudinal assessment, there is a critical need for a standardized, comprehensive, and reproducible echocardiographic protocol tailored to LVAD physiology. The aim of this paper is to provide a practical framework for routine ambulatory and inpatient echocardiographic assessment in patients with durable LVAD support, integrating standardized image acquisition and documentation with a structured interpretation of common pathological findings and device-related complications encountered in everyday clinical practice.

## 2. Basic Principles of LVAD Hemodynamics

In recent years, centrifugal continuous-flow LVADs have become the predominant form of durable mechanical circulatory support [1]. Axial-flow devices are no longer routinely implanted, and only a small number of patients remain supported by such systems. Since 2021, Medtronic™ has discontinued the distribution and sale of the HVAD system, and the vast majority of newly implanted devices are currently the HeartMate 3 centrifugal-flow LVAD by Abbott™. Accordingly, this article primarily focuses on contemporary centrifugal continuous-flow LVADs, with echocardiographic findings and hemodynamic principles largely referring to the HeartMate 3 system. However, these concepts are also broadly applicable to patients supported by the HeartWare HVAD.

Durable LVADs withdraw blood from the left ventricle and deliver it into the ascending aorta, thereby partially or fully bypassing native LV ejection. Contemporary devices provide no direct support to the right ventricle; therefore, adequate right ventricular (RV) function is mandatory to maintain left ventricular (LV) preload and effective LVAD flow.

LVAD performance is determined by

- **Pump speed;**
- **Preload** (RV function, volume status, pulmonary circulation);
- **Afterload** (systemic vascular resistance, arterial pressure).

Displayed LVAD flow is an estimated value, derived from pump speed, power consumption, and blood viscosity, and does not represent a direct volumetric measurement. Consequently, echocardiographic assessment of ventricular size, septal position, valve function, and forward stroke volumes are indispensable for physiological interpretation of LVAD parameters.

Changes in pump speed directly affect intracardiac pressures [3]. Increasing rotor speed reduces LV pressure and promotes unloading, often preventing aortic valve opening. Excessive unloading may result in leftward septal shift, impaired RV geometry, worsening tricuspid regurgitation, and RV failure. Conversely, inadequate unloading or increased afterload leads to elevated LV and left atrial pressures, pulmonary congestion, rightward septal shift, and secondary RV deterioration.

Understanding these interactions between the device and the native heart is fundamental for correct echocardiographic interpretation in LVAD patients.

## 3. Timing and Follow-Up Echocardiography in LVAD Patients

Echocardiography is the cornerstone of noninvasive follow-up in patients supported by left ventricular assist devices. Serial examinations are essential to monitor cardiac adaptation to mechanical support, assess device–heart interactions, and identify clinically relevant changes over time.

Following LVAD implantation, echocardiographic examinations are performed at short intervals depending on the clinical situation. After clinical stabilization, patients are assessed at least once weekly on the general ward. Shortly before hospital discharge, a comprehensive echocardiographic examination should be performed and carefully documented to serve as a reference baseline.

Thereafter, echocardiography should be performed at every scheduled outpatient visit at the LVAD center. Depending on local outpatient care structures, follow-up visits should occur approximately every three months during the first year after implantation and at least semiannually thereafter. In addition to scheduled examinations, echocardiography should be performed at every hospital admission—regardless of the reason for admission—and whenever clinical symptoms such as peripheral edema or exertional dyspnea occur as well as in the presence of LVAD alarms or relevant changes in device parameters, such as a progressive increase or decrease in estimated pump flow.

## 4. Parameters Displayed on the LVAD Console and Their Interpretation

The LVAD console displays the following parameters:

- **Rotation per minute (RPM)** refers to the speed the LVAD is working at—higher rpm ensures higher blood flow—which runs from 3000 to 9000 rpm in HeartMate 3™ devices.
- **Watt** refers to the energy use of the LVAD; it is an important parameter to consider, as changes in energy consumption may reflect pre-, intra- or post-pump obstructions [4].
- **LVAD Flow** is an estimated parameter derived from pump-specific algorithms based on rotor speed, power consumption, and the patient’s hematocrit. Typical flow values range from approximately 3.0 to 6.0 L/min.
- **Pulsatility index (PI)** evaluates the device’s performance and the patient’s hemodynamic status. A higher PI indicates more pulsatile flow, which is generally associated with better cardiac function, while a lower PI may suggest less effective circulation or potential complications.

## 5. Standardized Imaging Protocol

Echocardiographic evaluation in patients supported by mechanical circulatory support should follow a standardized approach. Such standardization is essential to improve reproducibility and to reliably document temporal changes during mechanical support. Only a structured and standardized assessment enables meaningful longitudinal comparisons and provides the basis for informed long-term device optimization and medical therapy in mechanical circulatory support (MCS) patients.

The imaging protocol should be grounded in the chamber quantification recommendations of the American Society of Echocardiography and the European Association of Cardiovascular Imaging [5] as well as in current consensus statements on multimodality imaging in patients with left ventricular assist devices and temporary mechanical support [6]. Serial echocardiography plays a central role not only in monitoring cardiac reverse remodeling [7] but also in assessing the potential for myocardial recovery and candidacy for LVAD weaning [8,9].

In every standardized examination, the type of LVAD, pump speed, estimated LVAD flow, pulsatility index, and blood pressure (mean arterial pressure in the absence of pulsatile flow) should be documented.

The following sections outline the key echocardiographic views and measurements and describe their interpretation in context. The imaging protocol presented here is intended to be applied during every routine echocardiographic examination of patients supported by an LVAD and, thus, serves as a standardized baseline assessment. As such, it is designed to be feasible not only in specialized LVAD centers but also in non-specialized clinical settings.

If this standardized assessment reveals findings that deviate substantially from the expected “normal” echocardiographic appearance (see Section 9), further advanced diagnostic procedures—such as ramp testing, transesophageal echocardiography, computed tomography, or right heart catheterization—may be required and should be performed in specialized centers. This is particularly relevant when echocardiographic abnormalities are accompanied by changes in LVAD parameters (see Section 4) or by clinical symptoms.

All required echocardiographic views are clearly illustrated in Supplementary Figure S1.

### 5.1. Parasternal View

Parasternal imaging of the heart is of particular importance in echocardiographic evaluation of patients with LVADs. While orthogonal apical imaging is often impeded by the device, parasternal image quality is usually preserved and allows for a wide range of views and measurements. The following echocardiographic views and recordings are recommended:

- Parasternal long axis view (2D, video);
- Parasternal long axis view (color Doppler aortic + mitral valve, video);
- Parasternal long axis view (M-mode of the aortic valve);
- RV inflow view (2D, video);
- RV inflow view (color Doppler tricuspid valve, video).
- Parasternal short-axis view:
  ○ Mitral valve level (video);
  ○ Mid-papillary level (video);
  ○ Aortic valve level (video);
  ○ Aortic valve Level (color Doppler aortic valve, video);
  ○ Aortic valve level (color Doppler pulmonary valve, video);
  ○ Aortic valve level (pw-Doppler right ventricular outflow tract, video);
  ○ Aortic valve level (color Doppler tricuspid valve, video);
  ○ Aortic valve level (cw-Doppler tricuspid valve, video).

### 5.2. Apical View

The device is positioned at the apex and usually prevents acquisition of the classic, apical four-chamber view of the heart. Instead, imaging from a more apical position using a slightly modified or foreshortened four-chamber approach is often feasible. This perspective is not suitable for volumetric assessment of the left ventricle. However, the mitral valve, tricuspid valve, and the free wall of the right ventricle can usually be visualized and assessed reliably. The two-chamber and three-chamber views are often only obtainable in a highly modified form and are usually of limited diagnostic value. Orthograde alignment of the aortic valve or the left ventricular outflow tract with spectral Doppler is generally not achievable. However, color Doppler imaging may still provide useful information. The following echocardiographic views and recordings are recommended:

- Apical four-chamber view (2D, video);
- Apical four-chamber view (color Doppler mitral valve, video);
- Right ventricular focused four-chamber view (2D, video);
- Right ventricular focused four-chamber view (color Doppler tricuspid valve, video);
- Right ventricular focused four-chamber view (cw-Doppler tricuspid valve, video)

### 5.3. Subcostal View

The subcostal view is essential for assessment of the size and respiratory variability of the inferior vena cava. In this view, the device outflow graft can often also be visualized. Using pulsed-wave Doppler, the characteristic flow signal can be obtained. The subcostal window may additionally be used to gain further information on the position of the interatrial and interventricular septa as well as to assess right ventricular function and tricuspid valve performance. The recommended echocardiographic subcostal views and recordings include the following:

- Longitudinal view of the vena cava inferior;
- Subcostal four-chamber view;
- Color Doppler of the hepatic veins;
- PW-Doppler of the hepatic veins.

## 6. Interpretation of the Echocardiographic Findings

Beyond acquisition of the above-mentioned views and measurement of the corresponding parameters, echocardiography in LVAD patients requires a fundamental understanding of individual parameters in the context of hemodynamics, cardiac function, and device performance.

### 6.1. Left Ventricular Size and Function

**Figure 1:**
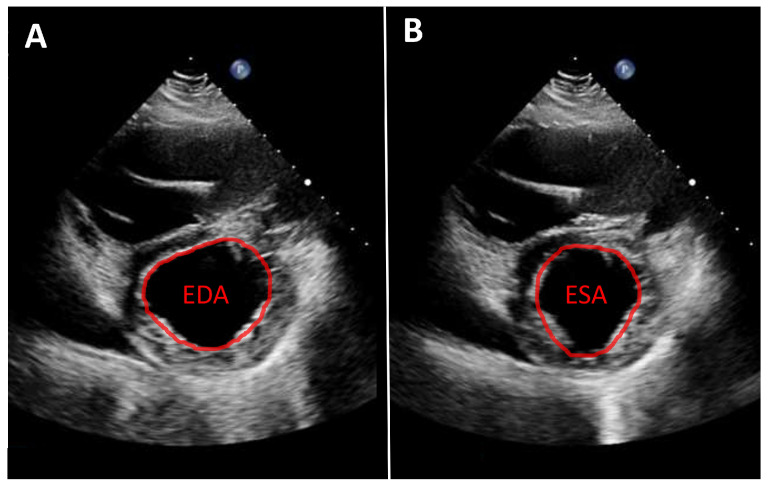
Parasternal short-axis view. Measurement of fractional area change (FAC) in a patient with an LVAD. (**A**): Planimetry of the end-diastolic area (EDA). (**B**): Planimetry of the end-systolic area (ESA). Calculation of FAC: FAC = (EDA − ESA)/EDA.

As noted above, left ventricular size should primarily be assessed using linear measurements. Implantation of a continuous-flow LVAD is typically associated with progressive left ventricular unloading and a corresponding reduction in ventricular size. While mean left ventricular end-diastolic diameter (LVEDD) reduction of approximately 1 cm has been reported, the magnitude of this change varies substantially between individual patients [7,10]. A substantial reduction in left ventricular diameter is often associated with a leftward septal shift and may occur in the context of excessive left ventricular unloading. Left ventricular function can likewise only rarely be quantified from apical views. Consequently, volumetric determination of ejection fraction as well as assessment of global longitudinal strain are usually not feasible. Therefore, linear measurements, such as fractional shortening, obtained from the parasternal long-axis view, are recommended. An even more accurate assessment, which is also frequently feasible, is measurement of left ventricular fractional area change in the parasternal short-axis view (Figure 1). Mild improvements in left ventricular function after LVAD implantation are frequently observed [7,10,11]. More substantial recovery of left ventricular function has been reported in a small but clinically relevant subset of patients [8,12,13]. In such cases, following comprehensive evaluation using dedicated testing and standardized weaning protocols, LVAD explantation may be considered.

### 6.2. Mitral Valve

Preoperative mitral regurgitation (MR) often decreases markedly after LVAD implantation [7,14]. Concomitant mitral valve repair at the time of LVAD implantation may be considered in selected cases but is not strongly recommended by current guidelines [15,16]. However, significant residual MR after LVAD implantation has been associated with persistent pulmonary hypertension and impaired right ventricular function [17,18].

Echocardiographic assessment of MR in LVAD patients should primarily rely on parasternal views, as apical imaging is frequently limited. In selected cases, apical visualization and quantification using the PISA method may be feasible. Importantly, the severity of MR is LVAD flow-dependent and influenced by LVAD settings. Only rare cases of percutaneous mitral valve repair (MitraClip™) after LVAD implantation have been reported [19].

### 6.3. Aortic Valve

In patients with an LVAD, aortic valve opening depends on left ventricular contractility, left ventricular filling, and the pump speed and resulting flow through the LVAD. A persistently non-opening aortic valve is not uncommon and is observed in approximately one third of patients [7]. A persistently non-opening aortic valve is associated with a poorer prognosis and promotes the development of aortic regurgitation [20,21,22,23]. For echocardiographic assessment of aortic valve opening, visualization in zoom mode as well as the use of M-mode are recommended. The degree of aortic valve opening should be documented in every examination. It has proven useful to distinguish between absent opening, intermittent opening, and regular opening of the aortic valve during the cardiac cycle.

Aortic regurgitation (AR) is a common finding in patients supported by durable LVADs. With contemporary continuous-flow devices, AR develops in approximately 8% of patients within the first year after implantation [24,25]. Its incidence increases progressively over time, with up to 25% of patients developing moderate or severe AR after 48 months, as reported in the Interagency Registry for Mechanically Assisted Circulatory Support (INTERMACS) registry [26].

The development of AR results in a recirculating loop between the aorta and the left ventricle, leading to ineffective forward flow and reduced systemic perfusion. Clinically relevant AR is associated with higher rates of heart failure-related hospitalizations and reduced survival [26].

**Figure 2:**
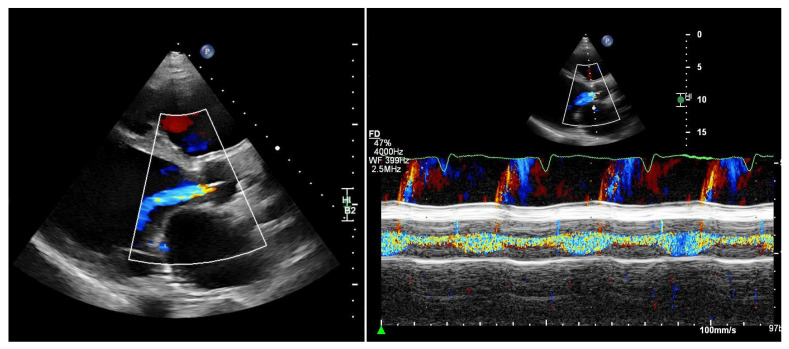
Patient with an LVAD and significant aortic regurgitation. Left: parasternal long-axis view. Right: M-mode of the aortic valve. M-mode color Doppler imaging clearly demonstrates both systolic and diastolic regurgitation.

Quantifying aortic regurgitation (AR) in patients supported by a left ventricular assist device (LVAD) remains challenging. Conventional echocardiographic parameters are based on the assumption of isolated diastolic regurgitation, an assumption that often does not apply in the LVAD setting. In fact, holocyclic regurgitation (systolic and diastolic) is frequently observed, particularly in patients with more advanced AR (Figure 2).

Consequently, accurate quantification of AR should ideally be performed at specialized centers. Comprehensive assessment typically requires transthoracic echocardiography supplemented by transesophageal echocardiography to improve visualization of the regurgitant jet as well as right heart catheterization for detailed hemodynamic evaluation [24,25].

As a practical and readily available approach, vena contracta width and the ratio of the regurgitant jet width to the left ventricular outflow tract (LVOT) diameter may be used for semi-quantitative assessment:

- Vena contracta width ≥ 3 mm;
- Jet/LVOT ≥ 46% (compared with ≥60% in native aortic valves).

Both parameters suggest at least moderate AR [27]. Conversely, if the regurgitant jet is absent or only mildly detectable during diastole, mild AR may be assumed. In addition, progressive left ventricular dilatation over time supports the presence of chronic volume overload and is consistent with hemodynamically significant AR, particularly in symptomatic patients.

More recently, a novel transthoracic pulse-wave Doppler-based parameter obtained from a modified right-sided parasternal window has been proposed. By interrogating the LVAD outflow graft, diastolic flow acceleration and the systolic-to-diastolic peak velocity ratio (S/D ratio) can be calculated [28]:

- Outflow graft S/D ratio < 5.0 indicates at least moderate AR;
- Diastolic acceleration > 49 cm/s suggests significant AR.

### 6.4. Right Ventricle

Even after LVAD implantation, the patient’s right heart must continue to function. Right ventricular contraction pumps blood through the pulmonary circulation, thereby ensuring adequate filling of the left ventricle and, consequently, sufficient inflow to the LVAD. Clinical experience shows that even ventricular fibrillation can be compensated for a limited period of time, as the LVAD is able to draw blood through the pulmonary circulation. However, the development of clinical decompensation—with peripheral edema, ascites, reduced exercise capacity, and ultimately declining LVAD flow—is an inevitable consequence over time.

Acute right ventricular failure after LVAD implantation occurs in approximately 10–40% of cases [29] and, in severe cases, may necessitate a right ventricular assist device. However, this entity is not the focus of the present review.

Chronic right ventricular failure is also a common phenomenon and manifests as clinical signs of right-sided heart failure, often requiring hospitalization and treatment with intravenous diuretics and/or inotropic therapy [30].

**Figure 3:**
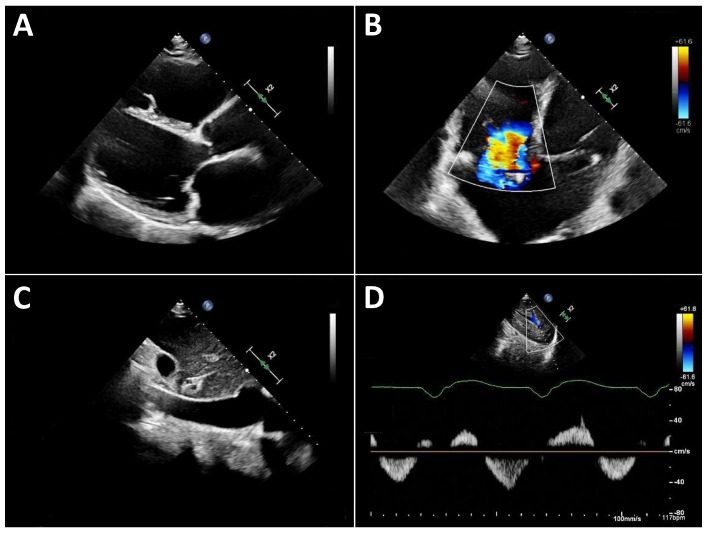
Typical echocardiographic findings of severe right heart failure in a patient with LVAD support. (**A**): Parasternal long-axis view showing a markedly dilated and hypokinetic proximal RVOT. (**B**): Apical view demonstrating pronounced right ventricular dilatation and severe tricuspid regurgitation. (**C**): Markedly dilated inferior vena cava with severely reduced respiratory variation. (**D**): Systolic flow reversal in the hepatic veins.

Typical echocardiographic signs of right ventricular failure include right ventricular dilatation, reflected by an increase in the proximal RV outflow tract (RVOT) diameter and RVD1 (Figure 3). This is frequently accompanied by dilatation of the tricuspid annulus with progressive tricuspid regurgitation. In severe cases, a leftward deviation in the interventricular septum may be observed. In advanced stages, the inferior vena cava is markedly dilated and exhibits significantly reduced respiratory variation. Quantification of tricuspid regurgitation can be performed using the standard guideline-recommended parameters, with a five-grade severity classification that has proven useful [31].

Three-dimensional echocardiography of the right heart is often not feasible in many LVAD patients due to limited acoustic windows. Therefore, assessment of right ventricular (RV) function primarily relies on two-dimensional parameters. Longitudinal RV function is frequently reduced after cardiac surgery [32], a finding that is also observed in patients with LVAD support. Accordingly, tricuspid annular plane systolic excursion (TAPSE) typically decreases after LVAD implantation [7,33]. This reduction in longitudinal function is, however, partially compensated by an increase in radial RV contraction.

Consequently, right ventricular fractional area change (FAC) is recommended over TAPSE for the assessment of RV function in LVAD patients [34]. Importantly, RV function under LVAD support cannot be adequately characterized by a single parameter. Instead, it should always be interpreted in the context of preload (inferior vena cava filling), afterload (pulmonary artery pressure), septal position (reflecting adequate left ventricular filling), and the severity of tricuspid regurgitation.

## 7. Interaction and Interdependence of Valve Function, Ventricular Function and Measured Parameters

There are substantial interactions between valvular dysfunction, ventricular function, and LVAD support. Isolated right ventricular failure, for example, rarely occurs in isolation; it inevitably leads to reduced left ventricular filling. As a consequence, the left ventricle may be unable to generate native systolic ejection, resulting in a persistently closed aortic valve and, ultimately, the development of secondary aortic regurgitation.

Another example is severe mitral regurgitation, which likewise leads to impaired effective left ventricular forward stroke volume and aortic valve opening promoting the long-term development of aortic regurgitation. In addition, by increasing pulmonary arterial pressure, severe mitral regurgitation may further contribute to right ventricular loading and dysfunction.

Adjustment of LVAD flow can have a substantial impact on echocardiographic parameters. For example, increasing LVAD flow by raising pump speed (RPM) alters intracardiac filling pressures [3]. This, in turn, affects cardiac geometry and the interaction between the right and left ventricles [35]. Therefore, achieving optimal device settings often requires the use of a ramp test, particularly in the presence of valvular regurgitation and/or right ventricular dysfunction. In such a ramp test, hemodynamics are assessed in parallel using right heart catheterization and echocardiography, while LVAD settings are adjusted according to a predefined protocol [36,37].

Details of ramp testing as well as specific protocols for evaluating myocardial recovery prior to LVAD explantation are beyond the scope of this review. Readers may refer to the relevant literature.

## 8. Interventions and Their Effects on Echocardiographic Findings

Various interventions may affect echocardiographic findings, and a clear understanding of these effects is essential for accurate interpretation and the initiation of appropriate measures.

**Increase in device speed:**

With increasing pump speed, LVAD flow increases, leading to higher aortic flow and increased total cardiac output to the systemic circulation. At the same time, left ventricular pressure decreases, resulting in reduced native ventricular ejection and less frequent and shorter opening of the aortic valve. As the pressure gradient between the left ventricle and the aorta is reduced, the severity of aortic regurgitation may increase. A reduction in left ventricular pressure may also lower pulmonary arterial pressure. At very high LVAD flows, leftward septal shift can occur, potentially contributing to worsening right ventricular failure.

**Decrease in device speed:**

With decreasing pump speed, LVAD flow and total cardiac output to the systemic circulation are reduced. Left ventricular filling and pressure increase, which may improve native ventricular ejection and provide sufficient myocardial function, resulting in more frequent and prolonged opening of the aortic valve. Concurrently, increasing left ventricular pressure leads to higher pulmonary arterial pressure, thereby increasing right ventricular afterload.

**Afterload:**

Modern centrifugal continuous-flow LVADs exhibit marked afterload dependency. Increased afterload results in a significant reduction in LVAD flow, whereas decreased afterload can lead to substantial flow augmentation. Significant afterload reduction, such as during overly aggressive antihypertensive therapy or septic shock may, therefore, cause excessive LVAD flow, with subsequent reduction in left ventricular pressure, diminished aortic valve opening, decreased pulmonary arterial pressure, and—in severe cases—leftward septal displacement.

**Preload:**

Adequate filling of the patient’s venous system is essential to ensure sufficient LVAD flow. The most common cause of device-related “low-flow” alarms is hypovolemia. Volume resuscitation increases right and left ventricular filling pressures, resulting in improved ventricular preload and a subsequent increase in LVAD flow, potentially accompanied by increased native ejection through the aortic valve. However, the right ventricle must be able to accommodate and pump the increased volume. In the presence of impaired right ventricular function and/or persistent valvular regurgitation, there is a risk of right ventricular decompensation.

An overview of potential interventions and their effects on hemodynamics and echocardiographic findings is provided in Table 1.

## 9. A Normal Echocardiographic Finding

With adequate interaction between the LVAD and the patient’s heart, the left ventricle may be markedly dilated. More important than ventricular size alone is the position of the interventricular septum, which should be midline and should not exhibit abnormal systolic leftward displacement throughout the cardiac cycle. Left ventricular systolic function may be severely reduced without representing a pathological finding in this context. In contrast, normal or near-normal ejection fraction (or fractional area change and fractional shortening) should prompt consideration of the potential for LVAD weaning, which should subsequently be evaluated in dedicated follow-up testing.

The aortic valve may remain closed throughout the entire cardiac cycle. However, particularly in patients without an intention for LVAD weaning or explantation, intermittent or regular opening of the aortic valve is generally preferable.

Ideally, no aortic regurgitation should be present. If aortic regurgitation is detected, it should be ensured that it is only mild. It should be noted that, due to their frequently continuous systolic and diastolic regurgitant flow, aortic regurgitation in LVAD patients is often underestimated when assessed using conventional echocardiographic parameters. Mild to moderate mitral regurgitation may occur and can usually be tolerated. In cases of severe mitral regurgitation, further evaluation should be considered, including a ramp test with right heart catheterization.

Evaluation of the right heart includes the conventional parameters recommended by current guidelines [38] (Mukherjee). However, TAPSE is frequently markedly reduced in LVAD patients and is, therefore, of limited value for routine assessment of right ventricular function. More useful global indicators of significantly impaired right ventricular function include right ventricular size, the severity of tricuspid regurgitation, and the diameter and respiratory variability of the inferior vena cava.

In the setting of an overall normal examination, only mild right ventricular dilatation and mild to moderate tricuspid regurgitation should be present. The inferior vena cava may be dilated as a consequence of long-standing cardiac disease, but preserved respiratory variability should still be demonstrable.

## 10. Summary

Echocardiography is an indispensable tool in the routine care of patients with durable LVAD support. A standardized imaging protocol, combined with a basic understanding of LVAD physiology and device–heart interactions, allows reliable assessment of cardiac function and hemodynamic status in everyday clinical practice.

For routine follow-up, a minimum echocardiographic dataset should include assessment of left ventricular size and septal position, documentation of aortic valve opening pattern, screening for relevant aortic and mitral regurgitation, and a global evaluation of right ventricular size, tricuspid regurgitation severity, and inferior vena cava diameter and respiratory variability. Interpretation of these findings must always be performed in conjunction with LVAD settings, preload and afterload conditions, and the overall clinical status of the patient.

The structured approach outlined in this review is intended to support sonographers and cardiologists in performing and interpreting routine echocardiographic examinations in LVAD patients, both in the outpatient setting and during non-device-related hospital admissions.

## Figures and Tables

**Table 1:** Hemodynamic interventions and their echocardiographic effects in LVAD patients.

Parameter	Hemodynamic Effect	Echocardiographic Findings	Potential Risks
Device speed ↑	LVAD flow ↑, LV pressure ↓, PA pressure ↓	Reduced aortic valve opening, LV size ↓, possible leftward septal shift	Aortic regurgitation ↑, RV failure due to septal shift
Device speed ↓	LVAD flow ↓, LV pressure ↑, PA pressure ↑	Increased aortic valve opening, LV size ↑	Decrease in total cardiac output, increase in RV afterload
Systemic Afterload ↓	LVAD flow ↑, LV pressure ↓	Aortic valve opening ↓, possible leftward septal shift	Suction events, RV dysfunction
Hypovolemia	LVAD flow ↓, intracardiac pressures ↓	Small LV cavity, reduced LV filling	Suction events
Volume administration	RV/LV filling ↑, LVAD flow ↑	Increased Aortic valve opening, RV and LV dilatation, increased tricuspid regurgitation	RV decompensation

LVAD, left ventricular assist device; LV, left ventricle; PA, pulmonary artery pressure; RV, right ventricle.

## Data Availability

No new data were created or analyzed in this study.

## Supplementary Materials

The following supporting information can be downloaded at https://www.mdpi.com/article/10.3390/jcdd13020070/s1, Figure S1: Standardized Echocardiographic Imaging Protocol for Patients with LVADs.

## Abbreviations

The following abbreviations are used in this manuscript:

LVAD	left ventricular assist device
VAD	ventricular assist device
RPM	rounds per minute
LV	left ventricle
RV	right ventricle
PI	pulsatility index
MCS	mechanical circulatory support
2D	two-dimensional
LVEDD	left ventricular end-diastolic diameter
MR	mitral regurgitation
AR	aortic regurgitation
INTERMACS	interagency registry for mechanical assisted circulatory support
LVOT	left ventricular outflow tract
RVOT	right ventricular outflow tract
TAPSE	tricuspid annular plane systolic excursion
FAC	fractional area change
