# Supplementary figures and images for: Routine Echocardiographic Assessment in LVAD Patients—A Structured Approach to Acquisition and Interpretation
Source: J Cardiovasc Dev Dis. 2026 Jan 30;13(2):70. doi: 10.3390/jcdd13020070 (PMC12942014; doi:10.3390/jcdd13020070)

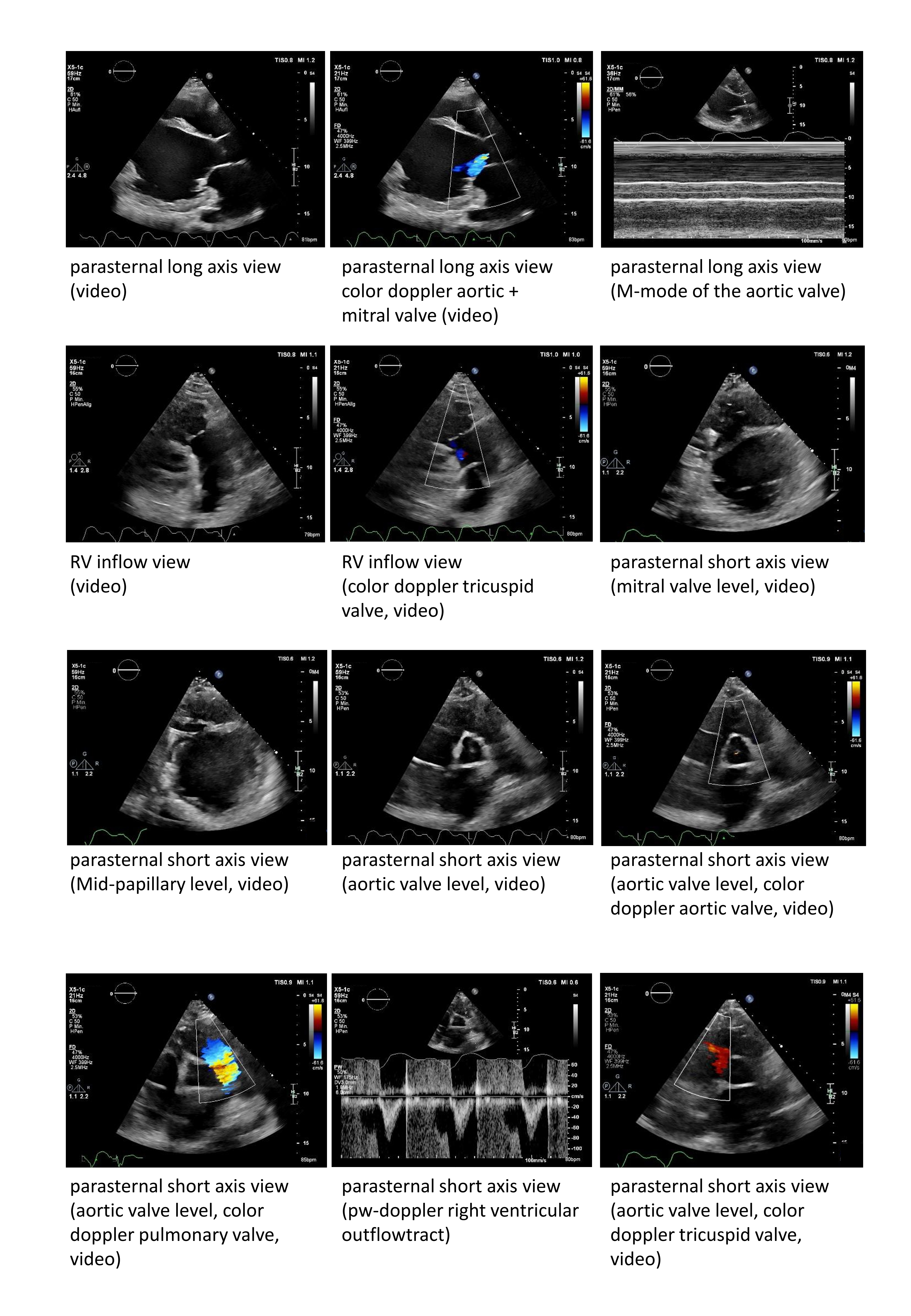

Supplement: Supplementary file 1 [file jcdd-13-00070-s001.zip › Supplement Figure 1A.jpg]

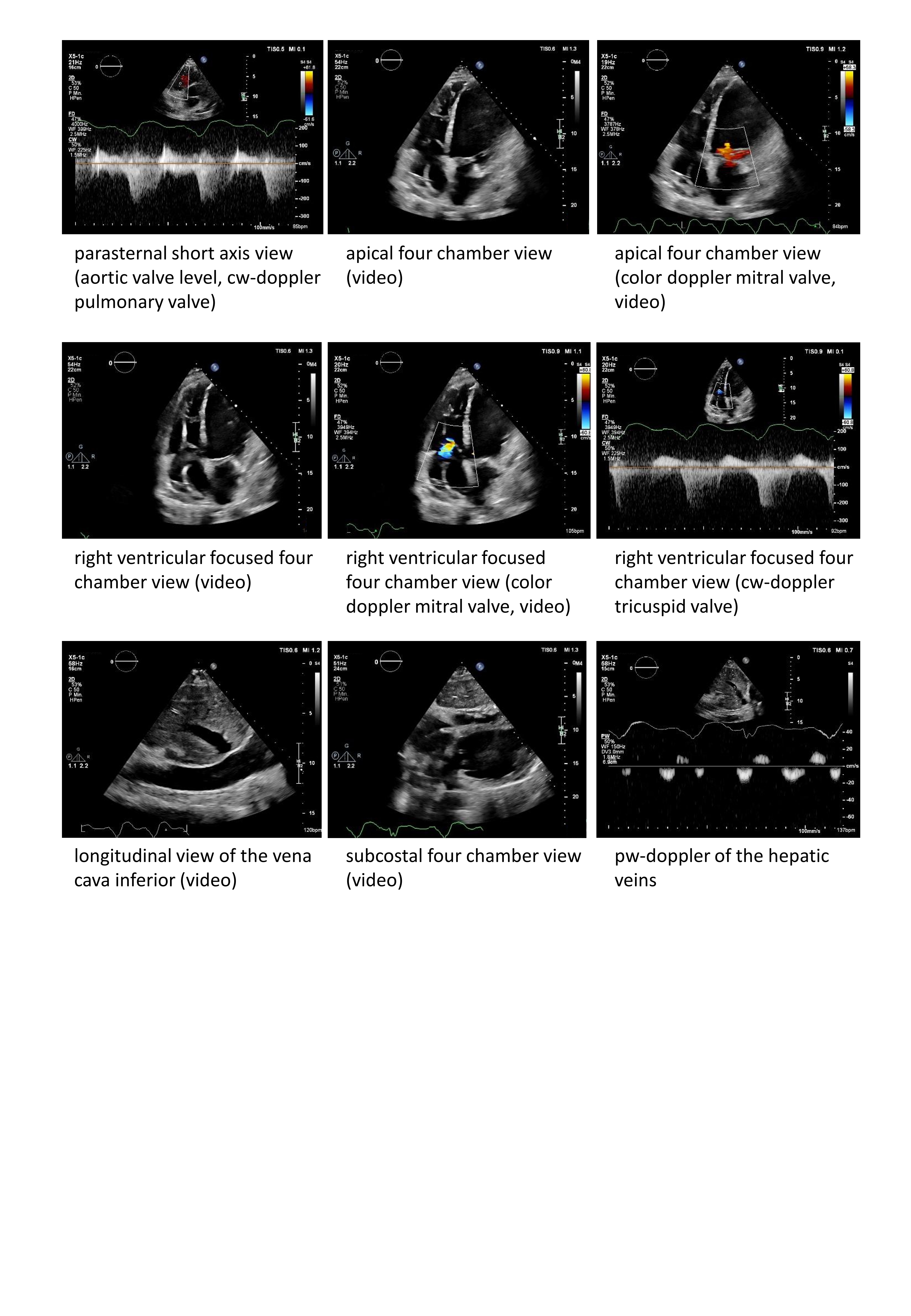

Supplement: Supplementary file 1 [file jcdd-13-00070-s001.zip › Supplement Figure 1B.jpg]
